# Volumetric Modulated Arc Therapy of the Pelvic Lymph Nodes to the Aortic Bifurcation in Higher Risk Prostate Cancer: Early Toxicity Outcomes

**DOI:** 10.1155/2015/696439

**Published:** 2015-10-11

**Authors:** Gina Hesselberg, Gerald Fogarty, Lauren Haydu, Nicole Dougheney, Phillip Stricker

**Affiliations:** ^1^Prince of Wales Hospital, High Street, Randwick, NSW 2031, Australia; ^2^University of New South Wales, High Street, Kensington, NSW 2052, Australia; ^3^Genesis Cancer Care, Mater Sydney Radiation Oncology, 25 Rocklands Road, Crows Nest, NSW 2065, Australia; ^4^University of Texas MD Anderson Cancer Center, Surgical Oncology, 1400 Pressler Street, FCT 17.6000, Houston, TX, USA; ^5^St Vincent's Hospital, 390 Victoria Street, Darlinghurst, NSW 2010, Australia

## Abstract

*Background*. Treatment of pelvic lymph nodes (PLNs) in higher risk prostate carcinoma is controversial. The primary focus of the study was to evaluate the early toxicity profile for this cohort of patients treated with Volumetric Modulated Arc Therapy (VMAT). *Methods*. Patient, tumour, and treatment characteristics of those who received VMAT from May 2010 to December 2012 were analysed. A simplified contouring process of the PLNs to the aortic bifurcation was developed based on consensus guidelines. Acute and late genitourinary (GU) and gastrointestinal (GI) toxicities were documented according to the Radiation Therapy Oncology Group (RTOG) Version 2 Guidelines. Successive Prostate Specific Antigen (PSA) values after treatment were measured on average 3 months apart. *Results*. 113 patients were treated between May 2010 to December 2012 with a median follow-up of 14 months. No patients experienced acute grade 3 or 4 GU and GI toxicity. Only 1 patient experienced a late grade 3 GU complication. No late grade 4 GU or GI events have yet occurred. *Conclusions*. This study reviews the first Australian experience of VMAT in the treatment of pelvic lymph nodes in prostate cancer, specifically to the level of the aortic bifurcation. It demonstrates a favorable acute toxicity profile whilst treating large PLN volumes with optimal dose coverage.

## 1. Introduction 

Prostate cancer is the most commonly diagnosed cancer in Australian males with an incidence of approximately 18,500 new cases per annum [1]. Management options for prostate cancer include radical prostatectomy (RP), radiotherapy (RT), androgen deprivation therapy (ADT), active surveillance, or a combination of these. The question of whether pelvic lymph nodes (PLN) should be treated in intermediate and high-risk settings with either surgery or radiotherapy is controversial. Two seminal phase III trials (RTOG 9413 and GETUG-01) reached conflicting conclusions [2, 3]. Additionally, no randomized trial has yet shown a survival advantage. Retrospective surgical series have demonstrated lower biochemical failure (BF) rates (defined as prostate specific antigen (PSA) greater than 0.2 ng/mL) in patients undergoing extended pelvic lymph node dissection [4]. This is applicable to patient populations with either clinically node negative disease [4] or low volume of nodal involvement [5]. Morikawa and Roach explore why some of these studies were negative in demonstrating a benefit of whole pelvic radiotherapy (WPRT) and conclude that predictions of nodal disease based on surgical series may in fact underestimate the true extent of involvement [6].

Consensus guidelines on pelvic lymph node clinical target volumes (CTV) in the setting of high-risk prostate cancer have been published to enable uniformity and accuracy in WPRT [7, 8]. Intensity modulated radiotherapy (IMRT) enhances treatment dose conformality [9]. This enables dose escalation to the clinical target volume whilst decreasing dose to surrounding normal tissue, thereby increasing the therapeutic ratio [9–11]. Volumetric modulated arc therapy (VMAT) has been shown in the Australian context to be superior to 3D conformal radiotherapy (3D-CRT) and step and shoot IMRT in terms of dose-volume histogram coverage of planning target volumes (PTVs) and organs at risk (OARs) [12]. Addintionally VMATis a further evolution of IMRT, enabling more efficient treatment [12]. VMAT can be utilized in the treatment of prostate cancer either in the definitive setting, in conjunction with high dose rate brachytherapy (HDRB) and post RP as either an adjuvant or salvage treatment. Compared to IMRT, VMAT is associated with lower rates of acute gastrointestinal (GI) and genitourinary (GU) toxicity in the treatment of prostate cancer [13].

This study documents the first Australian clinical experience of VMAT in the treatment of PLNs in higher risk prostate cancer. This occurred at the Mater Sydney Hospital, the Australian centre that has treated the largest cohort of such patients to date. The primary focus of the study was to validate our clinical impression of VMAT in terms of having an improved toxicity profile compared with published data on IMRT and 3D-CRT, particularly when treating large PLN volumes in the salvage setting following RP.

## 2. Materials and Methods

### 2.1. Patient Selection and Follow-Up

Patients with a diagnosis of prostate carcinoma who received VMAT radiotherapy from the start of the VMAT program in May 2010 to December 2012 were reviewed. Patient, tumour, and treatment characteristics were recorded and analysed. Staging details regarding the primary tumour, nodal involvement and presence of metastasis were derived from available documentation. The performance and extent of lymph node dissection was ascertained from the operation report or correspondence from the urologist. Due to the paucity of histopathological detail for patients who did not undergo RP, two separate cohorts were analyzed: those undergoing definitive RT and those who underwent adjuvant or salvage RT.

During treatment patients were assessed on a weekly basis. Acute and late genitourinary and gastrointestinal toxicities were documented according to the RTOG Version 2 Guidelines [14, 15]. Follow-up post treatment was performed at routine intervals, primarily by the treating radiation oncologist and if applicable, in conjunction with the referring urologist. The maximum toxicity suffered was recorded. Successive PSA values were measured on average 3 months apart. Given the short-term length of follow-up, oncological control was not a primary outcome of the study. An evaluation of early biochemical trends was performed by comparing the PSA levels before and after treatment as well as the need for ADT at one year following VMAT. Further analysis was performed to assess whether our clinical impression of patients who halved their PSA at 6 weeks following salvage radiotherapy continued to have a lowering of their PSA as observation continued.

### 2.2. Simulation

As per standard department policy, patients were requested to have an empty rectum and comfortably full bladder at simulation and treatment. CT simulation scans were performed in the supine position, scan window was from the top of L1 to mid femur, scanned at 2.5 mm intervals. Knee and feet supports and immobilization devices were utilized.

### 2.3. Contouring Technique

**Figure 1:**
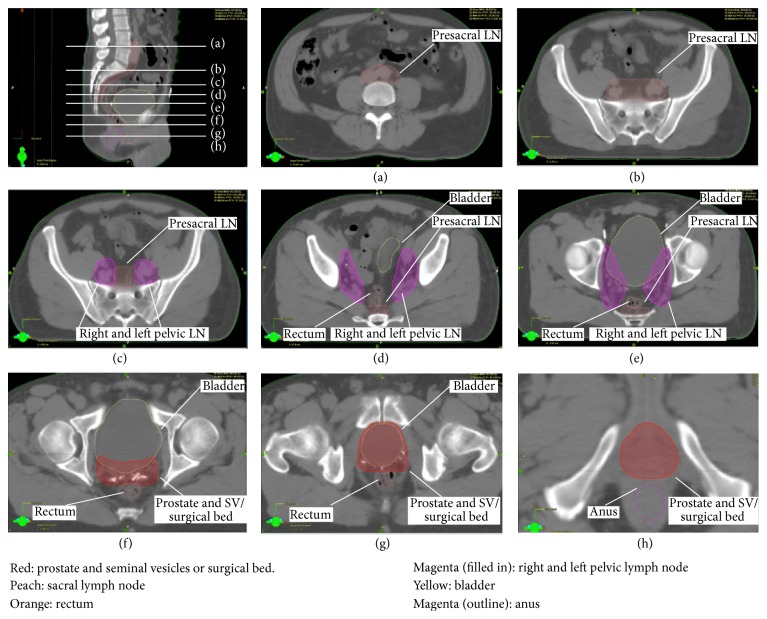
CT simulation scan demonstrating contoured volumes.

Contouring was manually performed by the treating Radiation Oncologist (GF). Clinical target volumes (CTVs) were contoured on the CT simulation scan with reference to RTOG and FROGG consensus guidelines [7, 8]. The prostate and seminal vesicles or the surgical bed of the prostate and seminal vesicles were contoured, with the aid of fiducial markers or surgical clips (Figures 1(f)–1(h)). The right and left PLN volumes were contoured, starting immediately above the prostate and seminal vesicle volumes (Figures 1(c)–1(e)). These volumes included the obturator, external, and internal iliac nodes with the anterior border beginning inferiorly at the anterior level of the acetabulum and following the external iliac artery posteriorly. The posterior border extended to encompass the internal iliac artery up to the bifurcation of the common iliac artery. The medial border of the volume was 0.5–1 cm short of the midline rectal structures. The right and left PLNs were combined into a single volume and treated as a single volume when no macroscopic nodal disease was present. The sacral lymph node volume started at the midline at the level of S3 (Figures 1(b)–1(e)). The contour was extended to embrace the bifurcation of the aorta, with the upper limit often at the level of the L4-5 disc space (Figure 1(a)). At the superior level of the previous right and left pelvic lymph node volumes, the sacral lymph node volume was expanded to include both the common iliac arteries. In the definitive, adjuvant, and salvage settings all of these volumes were expanded to a planning target volume (PTV) by 0.5 cm, excluding a volume termed “rectanus” (the combined contoured volumes of the anus and rectum). The anus was contoured from the first appearance inferiorly of a complete circle of sphincter tissue. The contour was taken in a superior direction until the most anterior circle that was devoid of rectal gas was reached (approximately 3-4 cm long). The rectum was then volumed superiorly from this level in a posterior direction until the structure started to turn anteriorly, which was taken as the start of the sigmoid colon. This was in concordance with the FROGG consensus guidelines [8]. The sigmoid colon and small bowel were also contoured but not excluded from the treatment volumes as these are structures on a mesentery and can therefore move between fractions. The dose volume constraints for each organ at risk are detailed in Table 1. The pelvic lymph node volumes were treated to higher doses if imaging or histopathology post RP showed disease in the pelvic lymph nodes. All patients were treated daily at five fractions per week. Image Guided radiotherapy (IGRT) with filming based on bony anatomy was done daily. A weekly kilovoltage CT scan was done on the department CT to confirm adequate bladder filling.

### 2.4. Treatment Planning and VMAT Delivery

**Figure 2:**
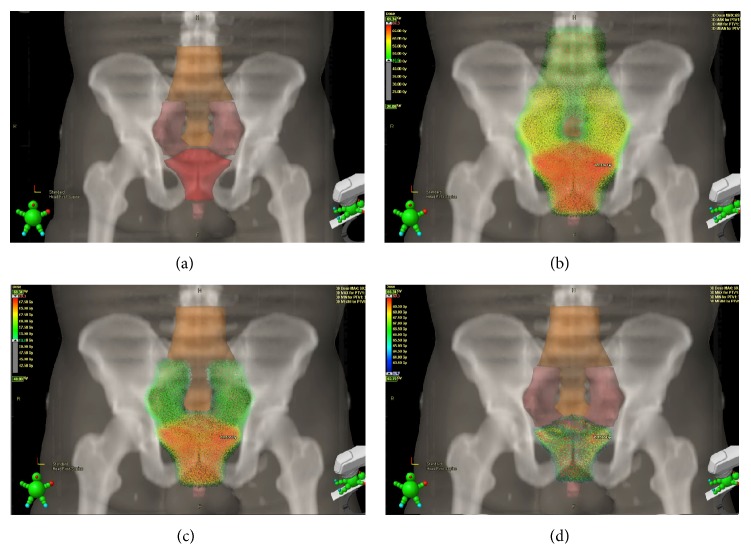
Dose distribution in adjuvant and salvage VMAT treatment. (a) Clinical target volumes: CTV 1 (orange) including presacral, common iliac, and para-aortic lymph nodes, left and right pelvic lymph nodes and prostate and seminal vesicles or prostatic fossa. CTV 2 (pink) including left and right pelvic lymph nodes and prostate and seminal vesicles or prostatic fossa. CTV 3 (red) including prostate and seminal vesicles or prostatic fossa. (b) Dose cloud superimposed on CTV 1: demonstrating dose of 49.5 Gy delivered to 95% of CTV 1 at 1.5 Gy per fraction for 33 fractions. (c) Simultaneous boost to 56.1 Gy: dose cloud superimposed on CTV 2 demonstrating dose of 56.1 Gy delivered to 95% of CTV 2 at 1.7 Gy per fraction for 33 fractions. (d) Simultaneous boost to 66 Gy: dose cloud superimposed on CTV 3 demonstrating dose of 66 Gy delivered to 95% of CTV 3 at 2 Gy per fraction for 33 fractions.

Treatment plans were generated using Eclipse version 8.6 (copyright Varian, Palo Alto). Treatment delivery was done using a 21ix Varian Linear Accelerator. VMAT was delivered in two to three arcs with maximum range of 360-degree with simultaneous variation of the gantry speed, dose rate, and leaf position. An energy of 10 MV and a max dose rate of 600 monitor units per minute were used. Treatment prescriptions are summarized in Table 2. Treatment was delivered using a simultaneous integrated boost (SIB) technique (Figure 2). Orthogonal kilovoltage images taken before the treatment confirmed patient position.

### 2.5. Statistical Analysis

The collected data was analysed to see if our clinical impression of patients who halved their PSA at 6 weeks following salvage radiotherapy continued to have a lowering of their PSA as observation continued. Statistical analysis was performed using IBM SPSS Statistic v21 (Chicago, IL) and SAS v9.3 (Cary, NC).

## 3. Results 

### 3.1. Patient and Tumour Characteristics

113 patients treated between May 2010 and December 2012 were identified. The median follow-up of the cohort was 14 months. Tables 3 and 4 summarize patient and tumour characteristics. Additional tumour characteristics for the cohort of patients who underwent RP are separated out in Table 4 due to the additional histopathological features available for this subset.

### 3.2. Toxicity

The acute GU and GI toxicity profiles for the entire and salvage cohorts are depicted in Table 5. Of note, no patients experienced an acute grade 3 or 4 complication. All acute reactions were symptomatically managed in the outpatient setting. No patients required hospital admission for management of acute side effects. In terms of late toxicity, only 1 patient experienced a late grade 3 GU complication. No late grade 4 GU or GI events have yet occurred at this early median follow-up.

### 3.3. Treatment Outcomes

**Figure 3:**
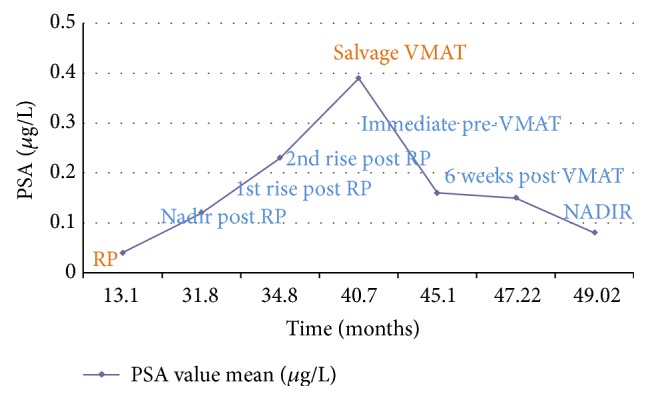
Average PSA dynamics following salvage VMAT treatment.

A subset analysis was performed on the PSA dynamics of the 38 patients who underwent salvage VMAT for biochemical failure following radical prostatectomy, excluding those patients who used ADT at any stage of their treatment. The mean nadir PSA level reached following VMAT was 0.08 ug/L at the end time point of this study. The PSA trend of biochemical failure following RP and the favorable trend following salvage treatment with VMAT is depicted in Figure 3.

In the same subset analysis of these 38 patients, analysis of the ratio of the PSA level taken immediately prior to salvage VMAT (defined as PSA_0_) and the PSA value at 6 weeks following salvage treatment (defined as PSA_6_) was undertaken. The mean PSA_0_ was 0.39 ug/L (range 0.04–7.9) and PSA_6_ was 0.15 ug/L (range 0.01–2.4). Twenty-one patients (55%) demonstrated a PSA_6_ : PSA_0_ ≤ 50% and 17 patients (45%) demonstrated a PSA_6_ : PSA_0_ > 50%. The relationship between the PSA_6_ : PSA_0_ ratio and BF following VMAT is demonstrated in Table 6. The sensitivity and specificity of PSA_6_ : PSA_0_ > 50% for determining biochemical failure was 80% and 60.6% respectively. The sensitivity and specificity of PSA_6_ : PSA_0_ > 75% for determining biochemical failure was 80% and 84.8%, respectively.

Five patients (13.2% of the salvage, no ADT cohort) demonstrated biochemical failure following their salvage VMAT treatment. One out of 21 patients with PSA_6_ : PSA_0_ ≤ 50% failed following their salvage VMAT treatment. The salvage treatment volumes for this particular patient only included the prostatic fossa as the patient had an extended lymph node dissection at the time of radical prostatectomy. This patient was retreated with a second course of salvage VMAT with lymph node volumes starting above his previous treatment level and extending superiorly to L4 and following this he remains biochemically disease-free. Of the 17 patients with a PSA_6_ : PSA_0_ > 50%, 4 patients demonstrated biochemical failure following their salvage treatment. Two of these 4 patients were treated with a second course of salvage VMAT to their upper pelvic lymph nodes, with treatment volumes starting above their initial salvage volumes. After the second course of salvage treatment, PSA levels demonstrated trends towards biochemical control (being 0.02 and 0.04 ug/L, resp.). The other 2 patients were investigated further with F-18 bone scans and found to have new bony metastasis in the ribs (*n* = 1) and spine (*n* = 1). One patient was subsequently commenced on ADT. This was the only patient out of the salvage cohort (3%) who went on to require ADT 12 months after their salvage treatment and PSA dynamics were excluded from analysis following commencement of ADT. The second patient who failed post salvage VMAT declined any further treatment at the time of his last review. Of note, ADT use 12 months following VMAT treatment for the entire cohort of patients was 12 out of 113 (11%).

## 4. Discussion

This study reviews the first Australian experience of VMAT in the treatment of pelvic lymph nodes of prostate cancer, specifically to the level of the aortic bifurcation. Our study audited 113 patients diagnosed with prostate cancer who were treated with VMAT at the Mater Hospital in Sydney. It demonstrates the utility of VMAT across a range of clinical indications. Moreover our results indicate a favorable acute toxicity profile whilst treating large pelvic nodal volumes with optimal dose coverage up to the level of the aortic bifurcation. Finally, our study intimates promising oncological outcomes as indicated by the PSA trend and minimal use of ADT post VMAT.

A particular focus of the study was to analyze the utilization of VMAT in treating PLN volumes in the salvage setting where a major concern is treatment morbidity given the large treatment volumes. The acute GU and GI toxicity profiles experienced by our salvage cohort can be compared to those reported in published data following treatment of pelvic lymph node volumes using different radiotherapy modalities. In a study by Alongi et al., the acute toxicity profiles of 172 patients who underwent adjuvant or salvage whole pelvis radiotherapy (WPRT) with either 3DCRT or IMRT were analyzed [16]. The median dose and dose range delivered to the pelvic lymph nodes in our study using VMAT and in Alongi's report on 3DCRT and IMRT were 52.8 Gy (46.1–66), 50.4 Gy (45–50.4), and 50.4 Gy (50.4–54.0), respectively [16]. With the use of 3DCRT, the reported lower and upper acute GI toxicities grade ≥ 2 were 8.6% and 22%, respectively, and acute GU toxicities grade ≥ 2 were 12.3%. In another study by Ashman et al., acute GI and GU toxicities grade ≥ 2 were reportedly as high as 57% and 34.7% [17]. Utilizing IMRT in WPRT delivers, as expected, an improved acute toxicity profile compared to 3D-CRT. Acute GI toxicities grade ≥ 2 have been reported as ranging from 6.6% to 40% and acute GU toxicities grade ≥ 2 ranging from 6.6% to 36.7% [9, 16, 18, 19]. Furthermore, studies have indicated that post-RP RT using IMRT is not associated with a decline in patient-reported urinary bowel or sexual quality of life indices at 2 years following completion of RT [20].

We have demonstrated in our study that with the use of VMAT, the acute toxicity profile can be improved upon even further. Acute GI and GU toxicities grade ≥ 2 for our salvage cohort were 34% and 13%, respectively. Similar promising results with VMAT have been reported in a recent study by Hall et al. in which acute GI and GU toxicities grade ≥ 2 were reported as 13.7% and 25%, respectively [13]. This observed benefit of VMAT may be due to its ability to deliver highly conformal dose distributions with improved target volume coverage and sparing of organs at risk [21]. This has been evident in the literature, which has demonstrated the superiority of both IMRT and VMAT in terms of dosimetry and sparing organs at risk compared to 3D-CRT [21–23]. VMAT further confers an additional advantage over IMRT and 3D-CRT in terms of its efficiency, safety, reduced monitor unit requirement, and cost-effectiveness [12, 22, 23]. VMAT delivered on treating a greater volume with an even better toxicity profile, further enhancing the therapeutic ratio in this small retrospective single institution study. Further follow-up of this cohort is required to ascertain whether a similar benefit is achieved in terms of late toxicities. Additionally, prospective randomized trials would be needed comparing the different radiotherapy modalities to conclusively demonstrate the toxicity profile advantage with VMAT.

Our study explored the validity of using the PSA value at 6 weeks post-VMAT treatment as a predictive tool for biochemical failure. Of the 21 patients who had a PSA_6_ : PSA_0_ ≤ 0.5, only 1 patient demonstrated BF with the remainder 95% of patients remaining free from biochemical failure. The sensitivity and specificity of PSA_6_ : PSA_0_ > 50% for determining biochemical failure was calculated as 80% and 60.6%, respectively. Our findings demonstrate that this parameter may be useful in predicting biochemical failure; however, the validity of this would need to be assessed in a more robust study design.

This audit demonstrates an easy and simplified contouring technique for whole pelvis radiotherapy including large nodal volumes up to the level of the aortic bifurcation. The technique used by the treating radiation oncologist in our study draws upon both the FROGG and RTOG consensus guidelines [7, 8]. Delineation of the surgical bed CTV was done as per the FROGG and RTOG guidelines.

There are several limitations of our study to acknowledge. Firstly, the retrospective nature of this audit made it prone to missing data. Investigator bias may exist in that the patients were treated by a single radiation oncologist at a single institution. Our median follow-up time is at this stage is insufficient to fully assess late toxicities and long term biochemical control. Additionally, our study lacks validated quality of life assessment tools. Finally, the superiority of VMAT over other treatment modalities would need to be assessed in a prospective randomized controlled trial.

## 5. Conclusions

VMAT can be utilized efficaciously in a variety of indications to manage carcinoma of the prostate especially in high risk disease where pelvic lymph node volumes can be included up to the aortic bifurcation. Our study demonstrates that this can be achieved with a favorable toxicity profile, both in the definitive and salvage settings. Short-term follow-up has demonstrated a trend towards favorable rates of biochemical control, which further supports the use of VMAT. With growing evidence to treat pelvic lymph nodes, both in the definitive and salvage settings, the utilization of VMAT will enable radiotherapy to be efficiently delivered to the required target volumes. Further follow-up is needed to assess long-term biochemical control and toxicity.

## Figures and Tables

**Table 1:** Dose-volume constraints for organs at risk.

Organ	Dose (Gy)	Volume (%)
Bladder	40	<60
Anus	40	<35
Rectum	40	<35
Rectum	50	<30
Sigmoid colon	40	<35
Bowel	45	<30
Penile bulb	40	<50
Femoral head	35	<100

**Table 2:** Dose, fractionation schedules, and treatment groups.

Treatment intent	Prescription dose	Number of fractions	Number of patients (%)		
			No ADT	ADT	Total
Definitive VMAT					
Intermediate risk	74 Gy	37	5	4	9
High risk	78 Gy	39	1	15	16
Total	—	—	**6 (5%)**	**19 (17%)**	**25 (22%)**
VMAT following HDR brachytherapy	50.4 Gy	28	9 (8%)	16 (14%)	25 (22%)
Salvage VMAT	66 Gy	33	38 (34%)	11 (10%)	49 (44%)
Adjuvant VMAT	66 Gy	33	9 (8%)	5 (4%)	14 (12%)
Total	—	—	62 (55%)	51 (45%)	113 (100%)

**Table 3:** Patient and tumour characteristics: entire cohort (*n* = 113).

Age (yrs)	Mean (range)	Distant metastases	*N* (%)
	67 (49–81)	No	107 (95%)
		Yes	4 (4%)
		Unknown	2 (2%)

T stage	*N* (%)	Gleason score	*N* (%)

T1-2a	38 (34%)	7	50 (44%)
T2b	8 (7%)	8	18 (16%)
T2c-4	60 (53%)	9	42 (37%)
Unknown	7 (6%)	Unknown	3 (3%)

Nodal status	*N* (%)	D'Amico Risk Group	*N* (%)

Negative	74 (65%)	Intermediate	46 (41%)
Positive	15 (13%)	High	63 (56%)
Unknown	24 (21%)	Unknown	4 (4%)

**Table 4:** Tumour characteristics: RP cohort (*n* = 63).

T stage	*N* (%)	Gleason score at margin	*N* (%)
T1-2a	20 (32%)	3	7 (11%)
T2b	1 (1%)	4	17 (27%)
T2c-4	37 (59%)	5	2 (3%)
Unknown	5 (8%)	Unknown	37 (59%)

Nodal status	*N* (%)	Seminal vesicle involvement	*N* (%)

Negative	44 (70%)	No	41 (65%)
Positive	9 (14%)	Unilateral	11 (17%)
Unknown	10 (16%)	Bilateral	5 (8%)
		Unknown	6 (10%)

Gleason score	*N* (%)	Vascular space involvement	*N* (%)

7	29 (46%)	No	40 (63%)
8	11 (17%)	Yes	15 (24%)
9	20 (32%)	Unknown	8 (13%)
Unknown	3 (5%)		

D'Amico Risk Group	*N* (%)	Perineural involvement	*N* (%)

Intermediate	31 (49%)	No	36 (57%)
High	29 (46%)	Yes	17 (27%)
Unknown	3 (5%)	Unknown	10 (16%)

Extracapsular extension	*N* (%)	Lymph node dissection (LND)	*N* (%)

Absent	19 (30%)	No	25 (40%)
Present	41 (65%)	Yes	37 (59%)
Unknown	3 (5%)	N/A or unknown	1 (1%)

Positive margin	*N* (%)	Extended LND	*N* (%)

Absent	34 (54%)	No	20 (32%)
Present	27 (43%)	Yes	22 (35%)
Unknown	2 (3%)	Unknown	21 (33%)

**Table 5:** Acute toxicity: entire and salvage cohorts.

	Grade	Entire (*n* = 113) number (%)	Salvage (*n* = 38) number (%)
Acute GU	0	21 (19%)	14 (37%)
	1	67 (59%)	20 (53%)
	2	25 (22%)	4 (10%)
	3 or 4	0 (0%)	0 (0%)

Acute GI	0	20 (18%)	4 (10%)
	1	62 (55%)	22 (58%)
	2	31 (27%)	12 (32%)
	3 or 4	0 (0%)	0 (0%)

**Table 6:** Biochemical failure (BF) post salvage VMAT (*n* = 38) and relationship to PSA_6_ : PSA_0_.

Number of patients with BF post-VMAT	5

Number of patients with BF post-VMAT and PSA_6_ : PSA_0_ > 0.5	4

% of patients with BF with PSA_6_ : PSA_0_ > 0.5	80% (4 of 5 patients)

% of patients with PSA_6_ : PSA_0_ > 0.5 with BF	23.5% (4 of 17 patients)

% of patients with PSA_6_ : PSA_0_ ≤ 0.5 **without** BF	95.2% (20 of 21 patients)

## List of Abbreviations Used

3D-CRT: 3D conformal radiotherapy
ADT: Androgen deprivation therapy
BF: Biochemical failure
CT: Computed tomography
CTV: Clinical target volume
EBRT: External beam radiotherapy
FROGG: Faculty of Radiation Oncology Genito-Urinary Group
GETUG: Genitourinary Tumor Group
GI: Gastrointestinal
GU: Genitourinary
HDRB: High dose rate brachytherapy
IMRT: Intensity modulated radiotherapy
PET: Positron emission tomography
PLN: Pelvic lymph nodes
PSA: Prostate specific antigen
PTV: Planning target volume
RP: Radical prostatectomy
RT: Radiotherapy
RTOG: Radiation Therapy Oncology Group
VMAT: Volumetric Modulated Arc Therapy
WPRT: Whole pelvic radiotherapy.

## References

[1] Ho W., Mills L., Negrello T., Min H., Connell E. (2012). *Cancer in Australia: An Overview 2012*.

[2] Roach M., DeSilvio M., Valicenti R. (2006). Whole-pelvis, ‘mini-pelvis,’ or prostate-only external beam radiotherapy after neoadjuvant and concurrent hormonal therapy in patients treated in the Radiation Therapy Oncology Group 9413 trial. *International Journal of Radiation Oncology Biology Physics*.

[3] Pommier P., Chabaud S., Lagrange J. L. (2007). Is there a role for pelvic irradiation in localized prostate adenocarcinoma? Preliminary results of GETUG-01. *Journal of Clinical Oncology*.

[4] Schiavina R., Bertaccini A., Franceschelli A. (2010). The impact of the extent of lymph-node dissection on biochemical relapse after radical prostatectomy in node-negative patients. *Anticancer Research*.

[5] Daneshmand S., Quek M. L., Stein J. P. (2004). Prognosis of patients with lymph node positive prostate cancer following radical prostatectomy: long-term results. *The Journal of Urology*.

[6] Morikawa L. K., Roach M. (2011). Pelvic nodal radiotherapy in patients with unfavorable intermediate and high-risk prostate cancer: evidence, rationale, and future directions. *International Journal of Radiation Oncology*.

[7] Michalski J. M., Lawton C., El Naqa I. (2010). Development of RTOG consensus guidelines for the definition of the clinical target volume for postoperative conformal radiation therapy for prostate cancer. *International Journal of Radiation Oncology Biology Physics*.

[8] Sidhom M. A., Kneebone A. B., Lehman M. (2008). Post-prostatectomy radiation therapy: consensus guidelines of the Australian and New Zealand Radiation Oncology Genito-Urinary Group. *Radiotherapy and Oncology*.

[9] Muren L. P., Wasbø E., Helle S. I. (2008). Intensity-modulated radiotherapy of pelvic lymph nodes in locally advanced prostate cancer: planning procedures and early experiences. *International Journal of Radiation Oncology, Biology, Physics*.

[10] Goenka A., Magsanoc J. M., Pei X. (2011). Improved toxicity profile following high-dose postprostatectomy salvage radiation therapy with intensity-modulated radiation therapy. *European Urology*.

[11] Riou O., Fenoglietto P., Laliberté B. (2012). Three years of salvage IMRT for prostate cancer: results of the Montpellier Cancer Center. *ISRN Urology*.

[12] Fogarty G. B., Ng D., Liu G., Haydu L. E., Bhandari N. (2011). Volumetric modulated arc therapy is superior to conventional intensity modulated radiotherapy—a comparison among prostate cancer patients treated in an Australian centre. *Radiation Oncology*.

[13] Hall W. A., Colbert L., Nickleach D. (2013). Reduced acute toxicity associated with the use of volumetric modulated arc therapy for the treatment of adenocarcinoma of the prostate. *Practical Radiation Oncology*.

[14] RTOG (2015). *Acute Radiation Morbidity Scoring Criteria*.

[15] RTOG (2015). *RTOG/EORTC Late Radiation Morbidity Scoring Schema*.

[16] Alongi F., Fiorino C., Cozzarini C. (2009). IMRT significantly reduces acute toxicity of whole-pelvis irradiation in patients treated with post-operative adjuvant or salvage radiotherapy after radical prostatectomy. *Radiotherapy and Oncology*.

[17] Ashman J. B., Zelefsky M. J., Hunt M. S., Leibel S. A., Fuks Z. (2005). Whole pelvic radiotherapy for prostate cancer using 3D conformal and intensity-modulated radiotherapy. *International Journal of Radiation Oncology Biology Physics*.

[18] Guerrero Urbano T., Khoo V., Staffurth J. (2010). Intensity-modulated radiotherapy allows escalation of the radiation dose to the pelvic lymph nodes in patients with locally advanced prostate cancer: preliminary results of a phase i dose escalation study. *Clinical Oncology*.

[19] McCammon R., Rusthoven K. E., Kavanagh B., Newell S., Newman F., Raben D. (2009). Toxicity assessment of pelvic intensity-modulated radiotherapy with hypofractionated simultaneous integrated boost to prostate for intermediate- and high-risk prostate cancer. *International Journal of Radiation Oncology, Biology, Physics*.

[20] Corbin K. S., Kunnavakkam R., Eggener S. E., Liauw S. L. (2013). Intensity modulated radiation therapy after radical prostatectomy: early results show no decline in urinary continence, gastrointestinal, or sexual quality of life. *Practical Radiation Oncology*.

[21] Teoh M., Clark C. H., Wood K., Whitaker S., Nisbet A. (2011). Volumetric modulated arc therapy: a review of current literature and clinical use in practice. *British Journal of Radiology*.

[22] Wolff D., Stieler F., Welzel G. (2009). Volumetric modulated arc therapy (VMAT) vs. serial tomotherapy, step-and-shoot IMRT and 3D-conformal RT for treatment of prostate cancer. *Radiotherapy and Oncology*.

[23] Palma D., Vollans E., James K. (2008). Volumetric modulated arc therapy for delivery of prostate radiotherapy: comparison with intensity-modulated radiotherapy and three-dimensional conformal radiotherapy. *International Journal of Radiation Oncology Biology Physics*.

